# Inner histopathologic changes and disproportionate zone volumes in foetal growth plates following gestational hypoglycaemia in rats

**DOI:** 10.1038/s41598-020-62554-2

**Published:** 2020-03-27

**Authors:** Vivi F. H. Jensen, Anne-Marie Mølck, Ingrid B. Bøgh, Jette Nowak, Birgitte M. Viuff, Charlotte L. M. Rasmussen, Louise Pedersen, Johannes J. Fels, Suzi H. Madsen, Fiona E. McGuigan, Pernille Tveden-Nyborg, Jens Lykkesfeldt, Kristina E. Akesson

**Affiliations:** 1 Novo Nordisk A/S, Department of Toxicology, Safety Pharmacology and Pathology, Maaloev, Denmark; 2 University of Copenhagen, Department of Veterinary and Animal Sciences, Section for Experimental Animal Models, Copenhagen, Denmark; 3 Lund University, Department of Clinical Sciences Malmö and Skåne University Hospital, Department of Orthopedics, Malmö, Sweden; 4 Novo Nordisk A/S, Department of Research Bioanalysis, Maaloev, Denmark

**Keywords:** Biological models, Gene expression analysis, Imaging, Microscopy, Biological techniques

## Abstract

Maternal hypoglycaemia throughout gestation until gestation day (GD)20 delays foetal growth and skeletal development. While partially prevented by return to normoglycaemia after completed organogenesis (GD17), underlying mechanisms are not fully understood. Here, we investigated the pathogenesis of these changes and significance of maternal hypoglycaemia extending beyond organogenesis in non-diabetic rats. Pregnant rats received insulin-infusion until GD20 or GD17, with sacrifice on GD20. Hypoglycaemia throughout gestation increased maternal corticosterone levels, which correlated with foetal levels. Growth plates displayed central histopathologic changes comprising disrupted cellular organisation, hypertrophic chondrocytes, and decreased cellular density; expression of pro-angiogenic factors, HIF-1α and VEGF-A increased in surrounding areas. Disproportionately decreased growth plate zone volumes and lower expression of the structural protein MATN-3 were seen, while bone ossification parameters were normal. Ending maternal/foetal hypoglycaemia on GD17 reduced incidence and severity of histopathologic changes and with normal growth plate volume. Compromised foetal skeletal development following maternal hypoglycaemia throughout gestation is hypothesised to result from corticosterone-induced hypoxia in growth plates, where hypoxia disrupts chondrocyte maturation and growth plate structure and volume, decreasing long bone growth. Maternal/foetal hypoglycaemia lasting only until GD17 attenuated these changes, suggesting a pivotal role of glucose in growth plate development.

## Introduction

It has previously been established that maternal hypoglycaemia may cause foetal growth restriction and in rodents, even short-term, maternal hypoglycaemia may disturb foetal skeletal development, reflected in decreased ossification and bone malformations^1–4^. The underlying mechanisms are, however, not clear; especially an understanding of the critical time windows of maternal hypoglycaemia and its duration to allow for normal versus abnormal foetal development is still lacking. We therefore established a model in non-diabetic rats to study the effect of sustained insulin-induced hypoglycaemia on foetal skeletal development^5^. Previously we showed that continuous maternal *hypoglycaemia throughout gestation* resulted in skeletal malformations, as well as decreased skeletal growth and bone mineral density^5^. In comparison *hypoglycaemia lasting only until GD17*, when organogenesis is completed, followed by normoglycemia allowed for near-normal development, emphasising that sufficient glucose supply late in gestation is crucial^6^.

To elucidate the principal mechanisms underlying the observed foetal skeletal defects, we have taken a wider approach to assess histological changes and to identify potentially regulatory pathways for bone growth and calcification. We hypothesised that hypoglycaemia disrupts regulation of the skeletal micro-environment by altering growth plate structure, as well as gene expression, and hormones essential for normal development of growth plates and bone cells, and that these changes would be more severe when *hypoglycaemia extended throughout gestation* compared with *only until end of organogenesis*.

The investigational strategy builds on our animal model of maternal hypoglycaemia induced by continuous infusion with human insulin (HI) lasting either throughout gestation until termination on GD20 or until GD17 (covering the embryonic period and organogenesis), followed by three days without insulin-infusion (GD17–20)^6^.

We used four main approaches: A descriptive histologic evaluation, quantitative histology, gene expression analysis, and finally, quantification of plasma hormones. The descriptive histologic evaluation consisted of recording any histopathologic disruption of growth plate organisation, including immunohistochemistry to visualise distribution of collagens, as well as in *situ hybridisation* to evaluate local expression of anti-hypoxic factors in growth plates. The quantitative assessment included stereologic measurement of growth plate volumes, spongious zone volume, and relative osteoblast: osteoclast volume in ossification centres. Gene expression analysis included quantification of genes related to growth plate structural composition, bone oxygen balance, the IGF-1 pathway, and osteoblast/osteoclast differentiation and activity. Major hormones known to influence bone metabolism such as corticosterone, IGF-1, and thyroid hormones were measured.

The data from this study will enable a better understanding of the pathogenesis of foetal skeletal changes resulting from continuous *hypoglycaemia throughout gestation* to GD20 (HI-*EoGest*) versus hypoglycaemia lasting only until *end of organogenesis* on GD17 (HI-*EoOrg*) in non-diabetic animals.

## Results

### Descriptive histology

#### Histopathologic evaluation

**Figure 1:**
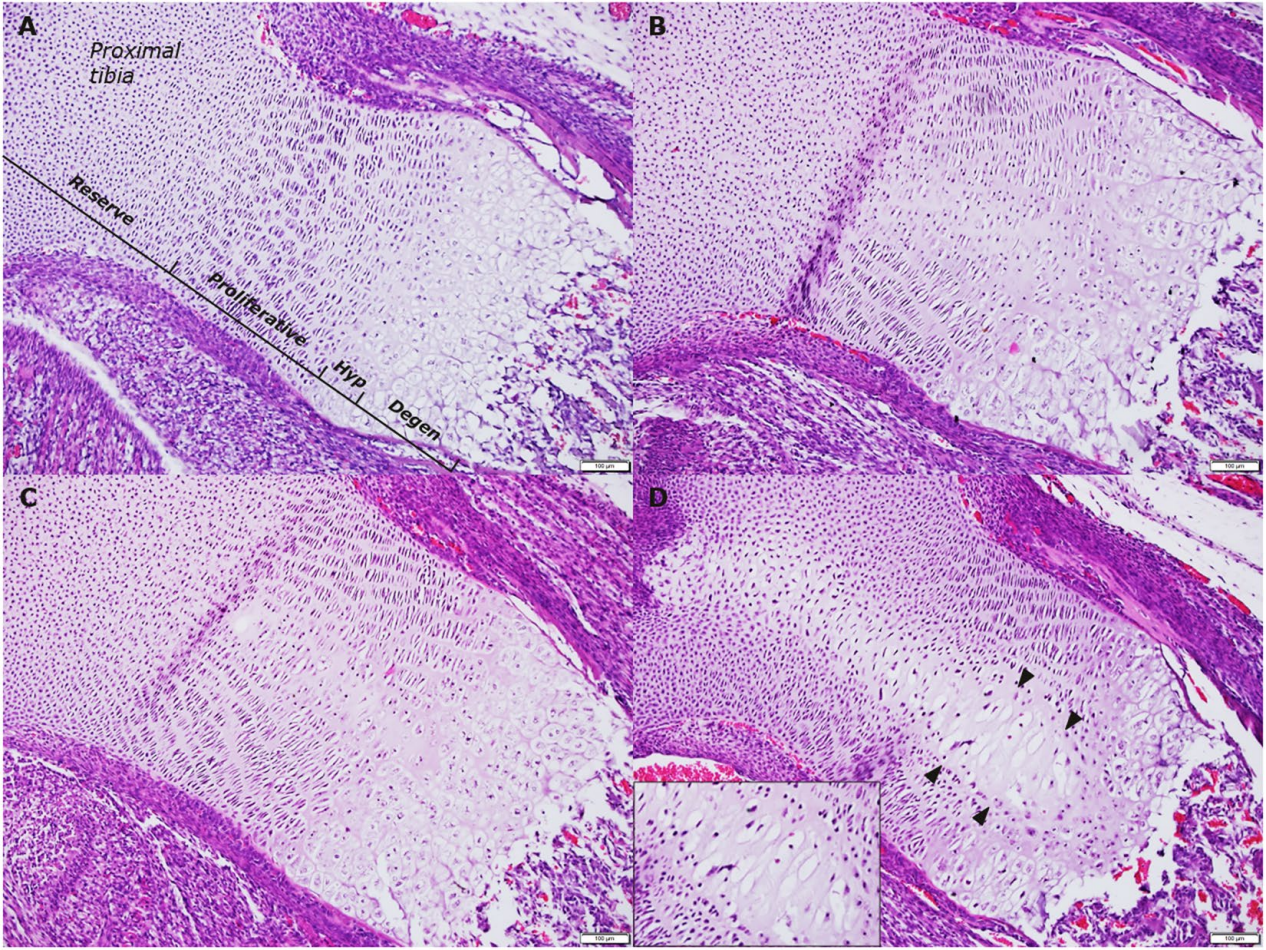
Representative examples of histopathologic changes in foetal tibial growth plates resulting from HI-infusion throughout gestation. (**A**) *Grade 0*, no changes. (**B**) *Grade 1*, minimal morphological changes centrally, involving all zones except reserve zone. (**C**) *Grade 2*, moderate morphological changes centrally, involving all zones except reserve zone. (**D**) *Grade 3*, severe morphological changes centrally (arrowheads), involving all zones, with loss of columnar organisation of chondrocytes, the few remaining chondrocytes appearing hypertrophic with pyknotic nuclei (insert). Degen, degenerative; Hyp, hypertrophic. Magnification: x100; insert x200.

Morphological changes localised the central longitudinal area of the growth plate were observed in the foetal tibial growth plates of the HI-infused groups (Fig. 1). All foetuses receiving HI-infusion throughout gestation (HI-*EoGest*), showed changes, which were *severe* and extended across all growth plate zones (Fig. 1d). The changes were characterised by fewer chondrocytes, which had lost their columnar organisation and displayed pyknotic nuclei and/or hypertrophy. Additionally, borders between the four growth plate zones (resting, proliferative, hypertrophic, degenerative) were irregular and less well-defined; particularly between the hypertrophic and degenerative zones and at the boundary between the degenerative growth plate zone and spongious zone of the primary ossification centre.

In contrast, when infused only until end of organogenesis (HI-*EoOrg*) approximately half the foetuses displayed histopathologic changes, and these were of *moderate* grade (Fig. 1c). Compared to CTRLs, the changes in the HI-*EoOrg* group were more pronounced.

#### Immunohistochemistry

**Figure 2:**
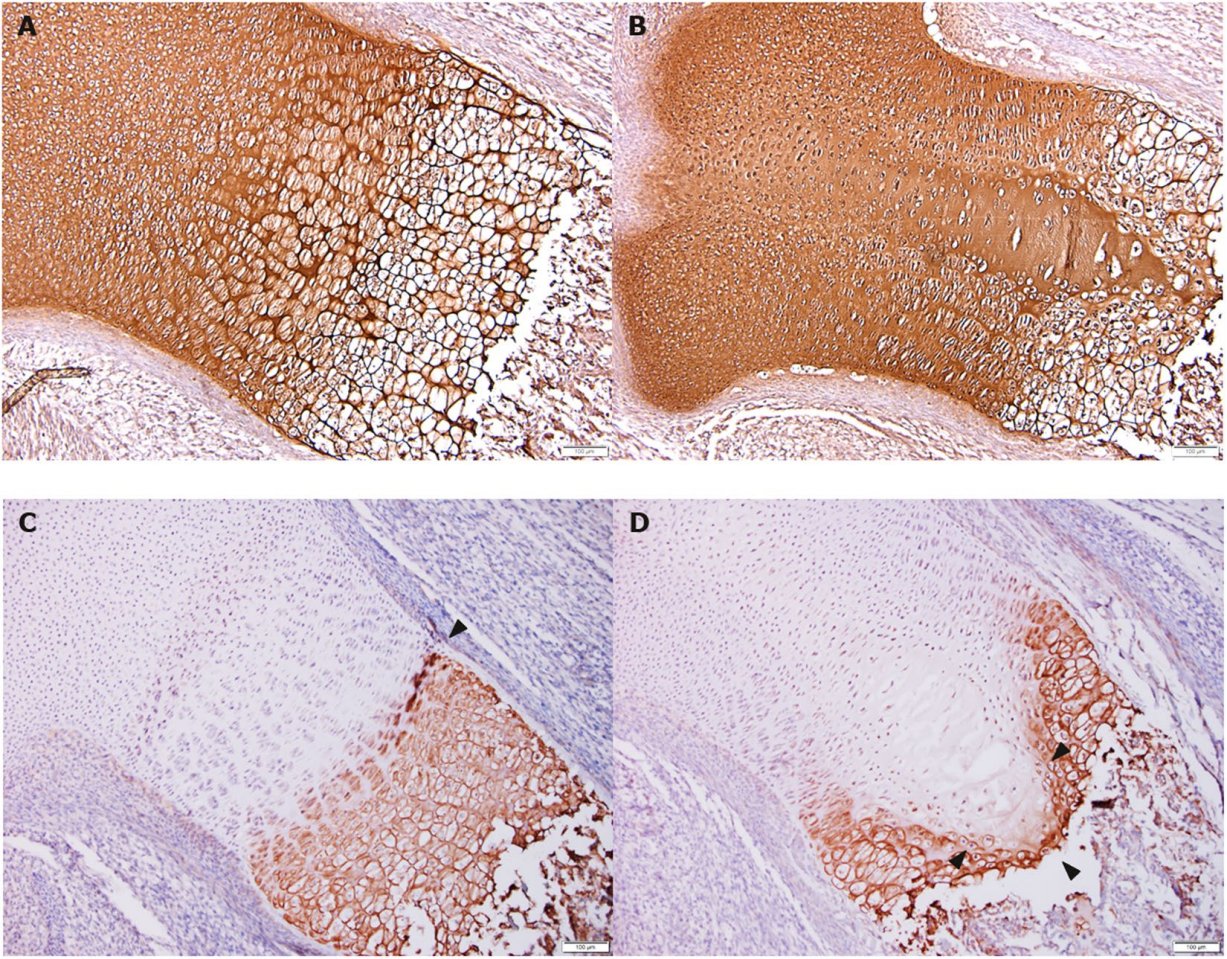
Immunohistochemical staining for collagen II (**A,B**) and collagen X (**C,D**) in foetal tibial growth plates. Sections stained for collagen II and X (dark brown). (**A**) CTRL: No histopathologic changes, normal distribution of collagen II throughout the growth plate, (**B**) HI-*EoGest*: Grade 3 changes, normal distribution of collagen II including the central area. (**C**) CTRL: No histopathologic changes. Peri-cellular collagen X staining between hypertrophic zone chondrocytes, clearly defining the boundary between proliferative and hypertrophic zones (arrowhead), (**D**) HI-*EoGest*: Grade 3 changes. Peri-cellular collagen X staining distally in the growth plate, no clear distinction of cell types and no central staining, resulting in a “V”-shaped staining pattern (arrowheads) pushing into hypertrophic, degenerative, and spongious zones. (Magnification: x100).

In controls, extracellular collagen II staining was observed throughout the growth plates (Fig. 2a); similarly, in the HI-*EoGest* group, staining was present in the central hypocellular area (Fig. 2b). In controls, collagen X distribution in growth plates clearly defined the boundary between proliferative and hypertrophic zones through positive staining of peri-cellular regions of hypertrophic chondrocytes (Fig. 2c). While the HI-*EoGest* showed a similar pattern, the cell types were not easily recognized, and the positive zone was markedly disordered (Fig. 2d).

#### *In situ* hybridization

**Figure 3:**
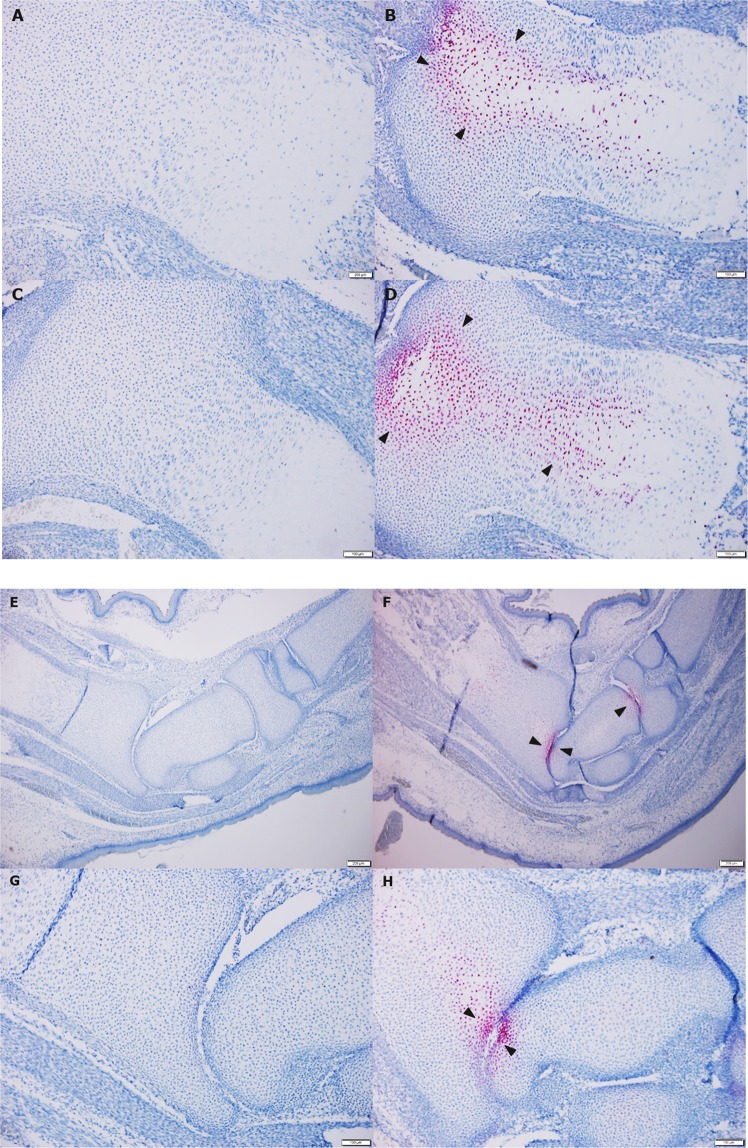
*In situ hybridization* for VEGF-A and HIF-1α in foetal tibial growth plates (**A–D**) and meta-tarsal joints (**E-H**). Sections stained for *VEGF-A and HIF-1α* (pink)*. Proximal tibial growth plate:* (**A**) CTRL: No histopathologic changes or staining, (**B**) HI-*EoGest*: Grade 3 changes and positive intracellular VEGF-A staining (arrowheads) in the cells at the periphery of the changes, (**C**) CTRL: No histopathologic changes or staining, (**D**) HI-*EoGest*: Grade 3 changes and positive intracellular HIF-1α staining (arrowheads) in the periphery of the changes. *Meta-tarsal joint*: (**E**) CTRL: No positive staining for HIF-1α, (**F**) HI-*EoGest*: Positive intracellular HIF-1α staining in articular cartilage (arrowheads), (**G**) CTRL: No positive staining for HIF-1α, (H) HI-*EoGest* - Positive intracellular HIF-1α staining (arrowheads) in articular cartilage. (A–D, G + H, x100 magnification; E + F, x40 magnification).

HI-*EoGest* foetal growth plates had pronounced positive staining for the pro-angiogenic factors HIF-1α and VEGF-A, locally restricted to the margin of the histopathologic changes (Fig. 3b,d). No positive staining was observed in controls (Fig. 3a,c), except for a few scattered positive cells in the degenerative zone and peri-articular cartilage in some of the control animals, which were not easily recognisable even at 100x magnification. Additionally, the peri-articular cartilage of foetal tibial and meta-tarsal joints displayed a markedly local increased HIF-1α expression (Fig. 3e–h). VEGF-A showed a similar expression pattern (data not shown).

As some basal expression of HIF-1α and VEGF-A is expected in growth plates, the lack of positive staining is likely due to the sensitivity being too low to detect this; however, since the staining was very marked in the cells surrounding the changes, showing a pronounced increase versus controls, this is not considered to significantly affect the results.

### Quantitative histology

#### Stereologic volume quantification

**Figure 4:**
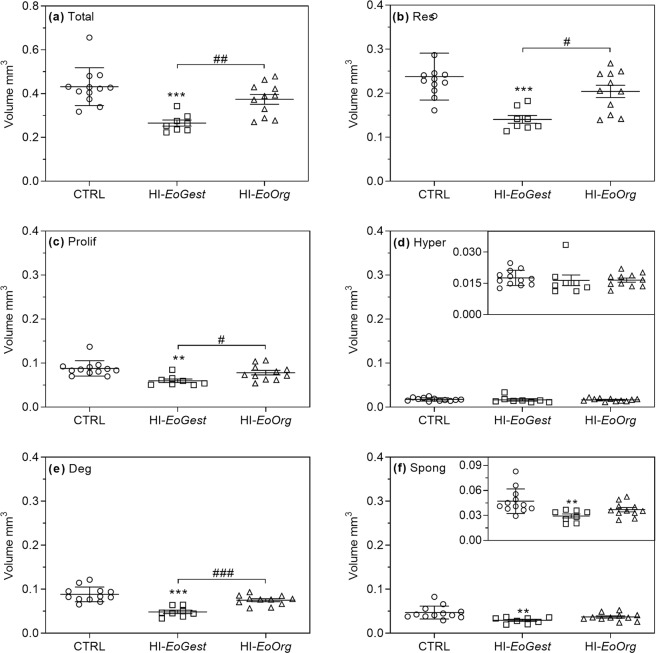
Absolute total growth plate and zone volumes in foetal long bones. Means ± SD and individual values. Absolute volumes of (**a**) Total growth plate, (**b**) (Res)erve zone, (**c**) (Prolif)erative zone, (**d**) (Hyper)trophic zone, (**e**) (Deg)enerative zone and (**f**) (Spong)ious zone. Inserts (**d,f**): Enlarged scale. **p < 0.01, ***p < 0.001 versus CTRL, ^#^p < 0.05, ^##^p < 0.01, ^###^p < 0.001 HI-*EoOrg* versus HI-*EoGest*. One-way ANOVA with *post hoc* Tukey’s multiple comparisons test.

**Figure 5:**
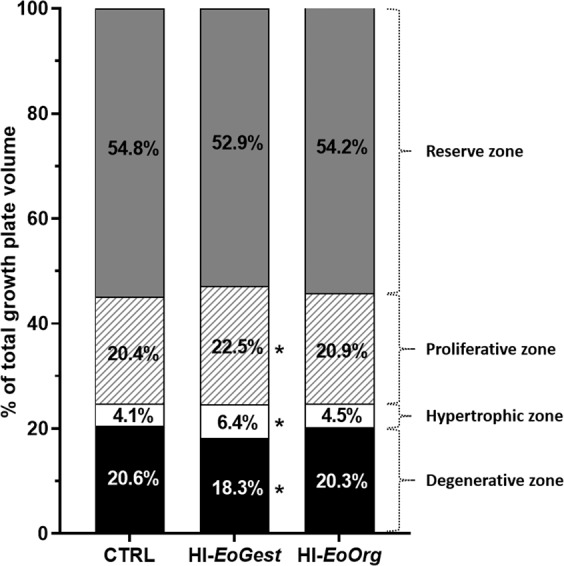
Relative volumes of tibial growth plate zones shown as percentage of total growth plate volume. Absolute zone volumes related to total growth plate volumes (group means). Each column represents 100% of the growth plates volumes. CTRL, n = 12; HI-*EoGest*, n = 8; HI-*EoOrg*, n = 11. *p < 0.05 versus CTRLs. One-way ANOVA with *post hoc* Tukey’s multiple comparisons test.

Growth plates. HI-infusion throughout gestation (HI-*EoGest*) resulted in decreased *total* growth plate volume (p = 0.0001, Fig. 4a) and reduced reserve, proliferative, and degenerative zone volumes (p < 0.0001–0.003, Fig. 4b–e). Disproportionately decreased total growth plate and zone volumes were reflected by increased *relative* volumes of the proliferative and hypertrophic zones (p = 0.0192; p = 0.0239), but conversely decreased the *relative* volume of the degenerative zone (p = 0.0436, Fig. 5). Recovering normoglycaemia after organogenesis (HI-*EoOrg*) resulted in increased *total* growth plate volume compared to the HI-*EoGest* (p = 0.0140), reaching control levels; similarly, the reserve, proliferative, and degenerative zone volumes did not differ from controls (p = 0.0007–0.0490). *Relative* zone volumes were in-between group HI-*EoGest* and CTRL levels.

Spongious zone. The *absolute* spongious zone volume was decreased in HI-*EoGest* animals (p = 0.0042), whereas volumes for the HI-*EoOrg* group ranged between HI-*EoGest* and CTRL groups (Fig. 4f). The *ratio* of spongious zone: total growth plate volumes was not affected by HI-infusion (mean ratios 0.10–0.11 ± 0.01–0.02 for all three groups; mean CE ≤ 0.05 and SD ≤ 0.01 for all).

Osteoblasts and osteoclasts. Immunohistochemical staining of osteoblasts nuclei/osteoclasts cytoplasm is illustrated in Supplementary Fig. S1. The *ratio* of osteoblast: osteoclast volumes in primary ossification centres was not affected by HI-infusion, regardless of duration (mean ratios CTRL 1.74 ± 0.32; n = 13, *EoGest* 1.71 ± 0.41, n = 7, HI-*EoOrg* 1.73 ± 0.34, n = 11; mean CE ≤ 0.02 ± 0.1 for all groups).

### Gene expression analysis

#### RT-qPCR quantification of foetal bone tissue mRNA levels

HI-infusion did not alter mRNA levels of cartilage collagens, hypoxia-related genes, the structurally related gene *comp*, genes related to the IGF-1 pathway nor osteoblast/osteoclast differentiation and activity (data not shown). In contrast, mRNA level of *Matn-3*, an additional structurally related gene, was decreased in HI-*EoGest* animals (p = 0.0222, data no shown), whereas recovering from hypoglycaemia after organogenesis (HI-*EoOrg*) resulted in normal mRNA level of this gene.

### Plasma hormone levels

#### Maternal and foetal corticosterone

**Figure 6:**
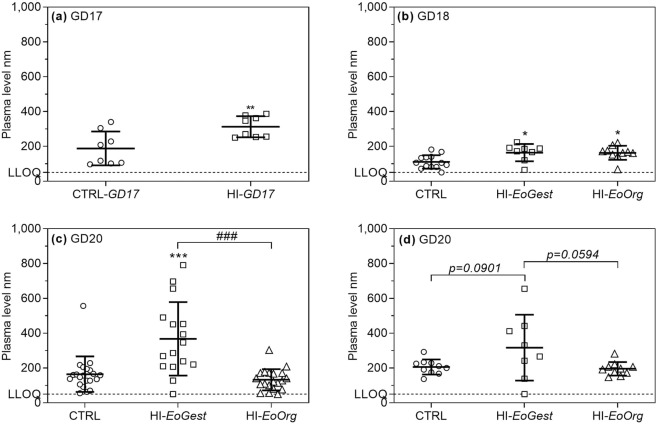
Plasma corticosterone levels: maternal (**a–c**) and foetal (**d**). Means ± SD and individual values. (**a**) Maternal levels at sacrifice on GD17. (**b**) Maternal levels on GD18. (**c**) Maternal levels at sacrifice on GD20. (**d**) Foetal litter levels on GD20. Dotted line: LLOQ (lower limit of quantification: 50 nmol/l). Four samples assayed <LLOQ and were set to 50 nmol/L (dams: 1 CTRL, GD18; 1 HI-*EoGest* and 1 HI-*EoOrg*, GD20. Litter: 1 HI-*EoGest*, GD20). *p < 0.05, **p < 0.01, ***p < 0.001 versus CTRLs. ^###^p < 0.001 HI-*EoOrg* versus HI-*EoGest*. (**a**) Unpaired two-tailed t-test, (**b–d**) One-way ANOVA with *post hoc* Tukey’s test.

*Maternal* corticosterone levels were increased in HI-infused animals on GD17 (HI*-GD17;* p < 0.0082) and GD18 (HI-*EoGest*, p = 0.0241; HI-E*oOrg* p = 0.0172. Figure 6a,b), with no difference between these groups. On GD20, HI-*EoGest* corticosterone levels remained elevated, whereas HI-*EoOrg* levels had returned to normal (p < 0.0001, Fig. 6c).

*Litter* corticosterone levels on GD20 differed between the three groups overall (p = 0.0478) but were not significantly different between individual groups (Fig. 6d). Exclusion of one HI-*EoGest* outlier (<LLOQ) strengthened the association (p = 0.0030) such that HI-*EoGest* levels were higher on GD20 compared to CTRLs and HI-*EoOrg* (p < 0.01).

As maternal and litter corticosterone levels in the HI-*EoGest* group were elevated on GD20, we tested the correlation between levels in individual dams and corresponding litter and this was strong (Pearson r = 0.834, p = 0.010, Supplementary Fig. S2).

#### Maternal and foetal IGF-1

*Maternal* IGF-1 levels were lower on GD17 (HI-*GD17* p = 0.0276, Supplementary Fig. S3). In contrast, on GD18 (24 h after infusion-stop in group HI-*EoOrg*), IGF-1 levels did not differ between groups though there was a trend for decreased levels in HI-*EoGest* (Supplementary Fig. S3).

On GD20, HI-*EoGest* IGF-1 levels were reduced (p = 0.0003, Supplementary Fig. S3), whereas after an infusion-free period from GD17-GD20 levels were normal (*HI-EoOrg*, p = 0.5417).

*Foetal* IGF-I levels were unaffected in HI-infused animals on GD20 (Supplementary Fig. S3).

#### Maternal thyroid hormones and TSH

*Maternal* TSH levels were unaffected by insulin-infusion (data not shown); likewise, for T_3_, although there was a trend towards increased levels in HI-*GD17* on GD17 (Supplementary Fig. S4). T_4_ levels were increased by HI-infusion (0.0009, Supplementary Fig. S4), a similar picture was seen 24 h later (GD18) (HI-*EoGest*, p = 0.0112; HI-*EoOrg*, p = 0.0064 versus CTRL. Suppl. Fig. 4). On GD20, HI-*EoGest* group T_4_ levels remained elevated (p < 0.0001), whereas HI-*EoOrg* group levels had returned to control levels (Supplementary Fig. S4).

## Discussion

We have previously used HI-infusion in a pregnant non-diabetic rat model to investigate the effects from *continuous hypoglycaemia* on foetal skeletal development. We showed that maternal *hypoglycaemia throughout gestation* delays foetal growth and skeletal development, while *hypoglycaemia only until after completion of organogenesis* (GD17) allows for near-normal development^6^. The mechanisms underlying these developmental changes are not fully understood. In order to elucidate this, we extended our investigation to assess for histopathologic changes and potential regulatory players in foetal bone development, based on the hypothesis that hypoglycaemia disrupts regulation of the skeletal micro-environment. The present study clearly shows that continuous *hypoglycaemia throughout gestation* causes pronounced histopathologic changes in tibial growth plates including disproportionately altered zone volumes. These changes are highly similar to changes caused by hypoxia, leading us to propose hypoglycaemia-induced hypoxia as a pivotal mechanism (Supplementary Fig. S5). When *hypoglycaemia lasted only until GD17*, histopathologic changes were less pronounced and growth plate volumes normal.

The observed severely disrupted ECM architecture in central foetal growth plates following *hypoglycaemia throughout gestation* may have resulted, at least in part, from decreased expression of the ECM protein MATN-3, essential to structural integrity of the chondrocyte scaffold^7^. In contrast, expression of COMP, collagen IX, and collagen II were unaffected, although similarly important for growth plate structural integrity through the formation of interconnected complexes with MATN-3^7,8^. In murine growth plates, ablation of any one of these proteins causes a longitudinal, central disruption of the columnar chondrocyte organisation and decreased cell numbers, occasionally accompanied by hypertrophic chondrocytes with pyknotic nuclei^9–13^. These changes are very similar to the appearance and confinement of the histopathologic findings in the current study and those in studies by others following growth plate hypoxia^14–17^, hence the rational for our hypothesis. Reversely, MATN-3 decreases may be secondary to a hypoxia-induced disruption of the ECM architecture. The less pronounced chondrocyte disorganisation and normal MATN-3 expression after *hypoglycaemia only until GD17* indicates that changes have either partly recovered after GD17 or the development has been attenuated. From this study, it cannot be concluded which of these scenarios take place, as growth plates were not evaluated on GD17.

In support of our interpretation, *extended hypoglycaemia* increased expression of the hypoxia-protecting factors, HIF-1α and VEGF-A, at the boundaries immediately surrounding the central histopathologic changes. Strikingly similar histopathologic changes were recorded in growth plates following ablation of either HIF-1α or VEGF-A in mice^14–17^ and *vice versa*, exposure of cultured chondrocytes to hypoxia increases expression of these factors^18–20^. Moreover, in mice, growth plate hypoxia induced by chondrocyte-specific inactivation of the HIF-1α gene increases VEGF-A expression at the periphery of the accompanying central growth plate changes^17^, similar to our findings. In line with this, histological growth plate changes were confined to the longitudinal central area, which is particularly vulnerable to hypoxia, being avascular and relying on passive oxygen diffusion from adjacent blood vessels^17,21^. This suggests that the histologic growth plate changes seen here are due to central hypoxia, which in turn elicits a local counter-regulatory increase of HIF-1α and VEGF-A levels.

On GD20, the increased maternal corticosterone levels following *hypoglycaemia throughout gestation* are likely caused by the hypoglycaemia, and is a known counter-regulatory response, increasing glucose-output from the liver^22^, also explaining changes in maternal IGF-1 and thyroid levels^23–25^. Since maternal and litter plasma corticosterone levels correlated in the hypoglycaemic animals, we speculate that increased maternal corticosterone level in turn exposed the foetus to increased levels through trans-placental transport^26^. Subsequently, foetal corticosterone may have impeded vascularisation in the growth plate adjacent to the metaphyseal area. Corticosteroid exposure has been shown to decrease production of VEGF-A by growth plate chondrocytes *in vitro* and *in vivo*^27,28^, and induce growth plate hypoxia by decreasing density of adjacent blood vessels in mice and piglets^27,29^. This is also in line with our observation of increased HIF-1α and VEGF-A mRNA levels in foetal tibial and meta-tarsal joint articular cartilage, which is equally avascular and vulnerable to decreased peri-articular angiogenesis following increased blood corticosteroid levels in mice^27,30,31^. The observed secondary increases in anti-hypoxia factors may be a compensatory response to corticosterone-induced hypoxia. In line with this, *ending hypoglycaemia on GD17* and normalising corticosterone levels may have interrupted the progression of the histopathologic changes.

In the above studies, no central histopathologic changes were observed in growth plates following corticosteroid exposure, however, this does not invalidate the present working hypothesis. Unlike the present study, administration was postnatal, and it is probable that the developing skeleton may be more vulnerable to corticosteroid exposure; counter-regulatory measures may also differ. Furthermore, temporary dosing was used, whereas in the present study, foetuses were exposed to high corticosterone levels, presumably, from conception.

*Hypoglycaemia throughout gestation* decreased total growth plate volume as anticipated based on previously observed shortened tibia length in litter-mates (foetuses from the same litters used for other investigations)^6^, which are known effects of glucocorticoid-dosing in rats and rabbits^32–34^. The disproportionate zone volume changes may be linked to reduced local VEGF-A levels. VEGF-A inactivation in growing mice expands the hypertrophic zone in tibial growth plates^35^, and chondrocyte-specific VEGF-A knock-out increases hypertrophic and decreases degenerative zone lengths in mouse embryo tibial growth plates (through delayed maturation/removal of hypertrophic chondrocytes)^15^. This is in line with the hypothesis of a key role of hypoxia in the present changes. Decreased MATN-3 expression may also be implicated, since MATN-3 KO promotes chondrocyte hypertrophy in mouse embryos^10,36^; this is reflected by an increased hypertrophic zone length in tibial growth plates following premature maturation of proliferative into hypertrophic chondrocytes^10^. In contrast to hypoglycaemia lasting *throughout* gestation, *hypoglycaemia ending on GD17* resulted in only slightly decreased total and separate growth plate volumes, which were not statistically different from controls; this was surprising given the shorter tibial length in litter-mates^6^. However, since this decrease in tibial length was less pronounced than after *hypoglycaemia throughout gestation*^6^, it possibly reflects delayed bone growth prior to GD17 extending beyond GD17, allowing only for an attenuation of the decrease.

In addition to a potential primary role of hypoxia in the development of the histopathologic growth plate changes, hypoglycaemia may also play an important role. The growth plate is a highly metabolic tissue, relying on anaerobic glycolysis^37^, and glucose serves as an essential source of energy^38^. Consequently, central chondrocytes may die due to glucose insufficiency and impaired glycolysis decreasing the energy supply. Apoptosis was seen despite local up-regulation of HIF-1α, which is known to increase expression of glucose transporters and enzymes of the glycolytic pathway^39–41^. However, this does not exclude a concurrent role of hypoxia in the changes; changes may be caused by a combination of hypoglycaemia and hypoxia.

Our data suggests that during foetal development, the growth plates are particularly vulnerable to maternal hypoglycaemia, more so than the ossification process, regardless of duration. High corticosteroid levels are known to decrease osteoblast and increase osteoclast activity and number/life-span in bone, leading to decreased bone formation and increased bone resorption ultimately reducing bone mineral density^30,42–44^. However, following *hypoglycaemia throughout gestation*, parameters relating to the bone mineralisation process did overall not change, in line with our previous findings of unaffected tibial peak mineral density in litter-mates in this group^6^.

Our investigation has some limitations; firstly, animal models are not necessarily translatable to humans, which should be kept in mind when interpreting the results. Moreover, pregnant diabetic women would not be continuously hypoglycaemic following insulin-dosing. Secondly, the rats are not diabetic, therefore the influence of counter-regulatory responses might differ in diabetics. This model was not intended as an animal model of diabetes, but rather, a model of experimentally insulin-induced hypoglycaemia, to be used as a means of understanding mechanisms involved in normal skeletal development.

This strengthens the study in several aspects including that it provides a stable controlled model in which all animals were similar at the outset and unaffected by disease. It also allows for investigation of changes to foetal skeletal development during hypoglycaemic conditions. Moreover, to better understand duration of continuous hypoglycaemia on foetal skeletal development we included both a group with insulin-induced hypoglycaemia *throughout gestation* as well as a group with *hypoglycaemia only until completion of organogenesis* (GD17) in order to also study prevention of foetal changes. Additionally, insulin-infusion was well tolerated and less stressful than the otherwise necessary multiple daily dosing needed to approach continuous hypoglycaemia, allowing for better animal welfare. Finally, the breadth of analytical methods employed provides linkage between observed histopathological changes and potential regulatory factors.

In summary, this experimental rat model of insulin-induced hypoglycaemia on foetal skeletal development shows pronounced effects on growth plate organisation; however, discontinuation of insulin-infusion after organogenesis allows for a partly normal development. We speculate that the skeletal changes are induced by hypoxia secondary to increased corticosterone levels (Supplementary Fig. S5). The hypoglycaemia induces a counter-regulatory increase in maternal plasma corticosterone levels, which in turn increases foetal corticosterone levels, decreasing oxygen supply to central growth plates, causing hypoxia, reflected by local counter-regulatory increases in anti-hypoxic factors and hypoxia-like histopathologic changes. Future follow-up investigations to validate this hypothesis could include measurement of tissue oxygen level in the growth plates, as well as histologic evaluation of: i) metaphyseal vascularisation (e.g. by immunostaining for endothelial cell markers), ii) local HIF-1α and VEGF-A protein levels (e.g. by immunohistochemistry), to determine if they mirror the mRNA changes, and iii) the apoptotic phenotype of the growth plate chondrocytes (e.g. by TUNEL assay).

In conclusion, our study underlines the importance of sufficient foetal glucose availability during late gestation for support of normal skeletal development.

## Materials and methods

### Experimental design

**Figure 7:**
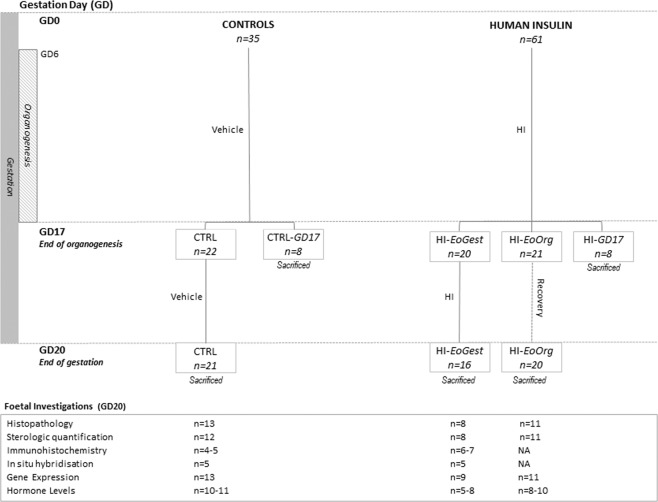
Study design including time-frames, investigations and animal numbers. Experimental groups received infusion with human insulin (HI) until end of gestation (EoGest) i.e. gestation day (GD) 20 (HI-*EoGest* group) or end of organogenesis (EoOrg) on GD17 (HI-*EoOrg* group). NA, not analysed.

The study design is presented in Fig. 7 and described in detail elsewhere^6^, but briefly, female Sprague-Dawley rats (approximately ten weeks old) were continuously infused with either HI or vehicle, starting one week prior to mating (Day 1).

The two experimental groups were: HI-infusion throughout and until end of gestation i.e. until gestation day (GD)20 (HI-*EoGest*); HI-infusion only until GD17 (i.e. approximate end of organogenesis) followed by a three-day infusion free period (HI-*EoOrg*). Control animals were infused with vehicle until GD20. Animals which died or were terminated prematurely were excluded; a total of 44 animals were infused with HI (16 received HI-infusion until GD20 and 20 until GD17); 29 were infused with vehicle. On GD17 eight animals from the HI-infused and control group were sacrificed for maternal blood sampling, these sub-groups were termed HI*-GD17* and CTRL-*GD17*, respectively. At the end of the study (GD20) all remaining animals were sacrificed, and various parameters assessed (described below).

It has been confirmed that insulin-infusion decreased maternal blood glucose levels as intended, from 3 h after infusion-start^6^; at the end of study/sacrifice HI-*EoGest* foetal blood glucose levels were also decreased.

All procedures involving live rats have been ethically approved by the Animals (Scientific Procedures) Act 1986 (ASPA) and United Kingdom Secretary of State and performed according to the ARRIVE guidelines, Directive 2010/63/EU, EC Commission Directive 2004/10, OECD Principles and Good Laboratory Practice, and The Good Laboratory Practice (Codification Amendments Etc.) Regulations 2004^45–48^, as well as Envigo and Novo Nordisk A/S company policies on the care and use of laboratory animals.

### Sampling of foetal tissue

Within one hour of sacrifice, one foetus per litter was taken from a mid-horn position (alternating between left/right uterine horn) from approximately half the dams in each group (Fig. 7). From each foetus, the left hind limb was fixed in 10% neutral buffered formalin and paraffin embedded for histology. The paraffin block was sectioned into 5 μm thick lateral longitudinal sections, mounted on glass slides, and stored at −20 °C until staining. The starting point was set at the first section where tissue appeared. From and including the first section, every fifth section was stained with haematoxylin and eosin (H&E) for histopathologic evaluation and quantification of growth plate zone volumes. The next slide in line after each of these sections was used for quantification of osteoblast-osteoclast ratios in the primary ossification centre. Sections were scanned (Aperio AT2 scanner, Leica Biosystems, Nussloch, Germany) and histopathologic evaluations and volume quantifications were performed blinded. From a subset of animals in the CTRL and HI*-EoGest* group (see below), the next three adjacent slides (i.e. third, fourth, fifth) were used for either immunohistochemistry or *in situ* hybridization (see below).

### Descriptive histology

#### Histopathologic evaluation

Foetal skeletal histopathologic changes were evaluated using a four-step grading scale based on severity and extent of changes (x10 and x100 magnification). Features for each grade were: *Grade 0*, no changes; *Grade 1*, minimal changes and only a slightly increased amount of extracellular matrix (ECM) across all zones, except the reserve zone; *Grade 2*, moderate changes, increased ECM and mild hypertrophy of chondrocytes across all zones, except the reserve zone; *Grade 3*, severe changes, characterised by increased though remarkably hypocellular ECM. The affected area extending through all growth plate zones.

#### Immunohistochemistry

To assess structural integrity and demarcation of the hypertrophic zone in tibial growth plates, distribution of collagen II and X was qualitatively evaluated by light microscopy from a subset of animals displaying severe (HI-*EoGest* group, n = 6–7 animals) or no changes (CTRL group, n = 4–5 animals). Representative sections, 2–4 sections per animal (slide 3, 4 or 5) were selected for evaluation. Immunohistochemistry was not performed in HI-*EoOrg* animals.

To identify osteoblasts and osteoclasts in primary ossification centres, tibial sections were stained for osterix (in osteoblast nuclei) and CD68 (localised in lysosome membranes of osteoclasts). Protocols are included in Supplementary Material.

#### *In situ* hybridization

To evaluate hypoxia-related changes in foetal growth plates, distribution of anti-hypoxic factors HIF-1α and VEGF-A was assessed in 2–4 representative sections from animals with severe or no changes (i.e. HI-*EoGest* and CTRL groups; 5 animals/group). *In situ* hybridization was not performed in HI-*EoOrg* animals. mRNA signal was detected using Automated Assay for Ventana Systems. The assay RNAscope 2.5 VS Reagent Kit-RED (322250,) Probe-Rn-Hif1a (432289) and Probe-Rn-Vegfa (315369) from Advanced Cell Diagnostics, Inc., Newark, CA, USA.

### Quantitative histology

#### Stereologic quantification of volumes

The sampling method described above resulted in 11–22 H&E stained sections per foetus that contained growth-plate. Volumes of each growth plate zone and adjacent primary spongious zone were quantified based on the Cavalieri principle^49^. Demarcation of individual zones was defined according to the morphologic criteria of chondrocytes in each zone described by Burdan *et al*.^50^. NewCAST software (Visiopharm, Hoersholm, Denmark) was used; point-counting was based on preliminary counting on randomized sections, with a minimum 150–200 hit-points in total and a coefficient of error (CE) < 10%. Grid systems used 7 × 8 (reserve zone) and 12 × 12 points (remaining four zones) per view field (on-screen magnification x100). The sampling fraction was 100% of growth plate area on each section (shape factor 4, smooth organ). For each zone, volume and mean CE were calculated according to stereologic principles^49,51^.

The primary ossification centre was included in 6–18 sections/foetus. Osteoblast nuclei and osteoclast cytoplasm total volumes were estimated using a grid system with 2 × 2 and 5 × 5 points per view field (on-screen magnification x400). Sampling fraction was 100% of the primary ossification centre per section (shape factor 6, smooth organ). Volumes and mean CE were calculated as above followed by calculation of volume ratio.

### Gene expression analysis

#### RT-qPCR quantification of foetal bone tissue mRNA levels

One foetus per litter was taken from a mid-horn position from the uterine horn (opposite to the above), hind limbs were sectioned at the hip-joint, soft tissue removed by microdissection, bones placed in aluminium foil envelopes, snap frozen in liquid nitrogen, and stored at −20 °C until RNA isolation. Total RNA was extracted using Trizol (15596026, Thermo Fisher Scientific, Waltham, MA, USA), purified using RNeasy Mini Kit (74104, QIAGEN AB, Sollentuna, Sweden), and reverse transcribed to cDNA using High-Capacity cDNA reverse Transcription Kit (4374966, Thermo Fisher Scientific). mRNA levels were quantified by Real-Time PCR using QuantStudio 7 Flex Real-Time PCR system and Taqman Gene Expression Assays (Applied Biosystems, Foster City, CA, USA).

Genes investigated included: *Col10a1*, *Col9a1, Col2a1, Comp, Matn-3* (for structural composition of cartilage/growth plate); *Hif-1α*, *Vegf-A* (for regulation of oxygen balance); *Trap, Alp, Runx2, Rankl (*for osteoblast/osteoclast differentiation and activity); *Igf-1, Igf-R, Igfbp-3, Igfbp-5 (*IGF-1 pathway). Housekeeping genes: *Gapdh* and *β-actin*.

Triplicate measurements were performed. If the standard deviation (SD) of mean C_T_ values exceeded 0.167 and one measurement was a clear outlier, this was removed. If there was no clear outlier, C_T_ values from all three measurements were included in the mean. The “∆∆C_T_ Method” was used to calculate relative expression of each target gene. First the target gene C_T_ value (C_T_, target) was normalized to the mean C_T_ value of the two housekeeping (reference) genes, (C_T_, ref), for each animal and target gene separately (∆C_T_ = C_T_, target-C_T_, ref). Thereafter, mean ∆C_T_ and SD were calculated for each target gene within each group. The ∆∆C_T_ value was then calculated by normalizing the group mean ∆C_T_ for each test group to the mean ∆C_T_ of the control group (∆∆C_T_ = mean ∆C_T_, test group - mean ∆C_T_, control group). Data are reported as −∆∆C_T_ values.

### Quantification of hormones in plasma

Sampling of blood for quantification of plasma hormone levels was performed on the following days:

*Maternal:* on GD17, groups CTRL-*GD17* (n = 8) and HI-*GD17* (n = 8) were sacrificed and sampled. On GD18, CTRL, HI-*EoGest*, and HI-*EoOrg* groups were sampled (approximately 24 h after infusion-stop in the HI-*EoOrg* animals). On GD20, at sacrifice, all dams and litters (pooled blood) were sampled.

#### Plasma corticosterone

Plasma corticosterone (maternal and foetal) was quantified using a commercial ELISA kit (EIA-5186, DRG International, Springfield, New Jersey, USA). Plasma was stored at −20 °C before analysis. Lower limit of quantification (LLOQ): 50 nM.

#### Plasma insulin-like growth factor 1 (IGF-1)

Plasma was pre-treated with a proprietary “releasing agent” to displace all IGF-1 bound to binding protein. IGF-1 (maternal and foetal) was quantified using a commercially available ELISA kit (E25, Mediagnost, Reutligen, Germany). LLOQ: 0.029 ng/ml.

#### Plasma thyroid hormones and thyroid stimulating hormone (TSH)

Plasma thyroid hormone (T_4_ and T_3_ - maternal) and TSH (maternal and foetal) was quantified using commercially available assays (STTHMAG-21K; Milliplex MAP Rat TSH Pituitary Panel (RPTMAG-86K, both Millipore (U.K) Ltd, Hertfordshire, UK). LLOQ: T4 (1.95 ng/ml), T3 (0.391 ng/ml) and TSH (123 pg/ml). Measurements were performed in duplicate. Reported means are from individual dams/litters.

### Statistics

#### Histopathology

Incidence and severity of histopathologic changes were compared between groups using Fishers exact test.

#### Stereology

In each animal total growth plate volume was calculated as the *sum* of each zone (reserve, proliferative, hypertrophic, degenerative). Also calculated for each zone were: the volume of each zone *relative* to total growth plate volume; the ratios of spongious zone: growth plate and osteoblast: osteoclast volume. Group means were compared using one-way ANOVA.

#### qPCR

For each target gene, ∆∆C_T_ values were compared using one-way ANOVA.

#### Plasma hormone levels

Comparisons between groups were analysed using one-way ANOVA unless more than one sample/group was <LLOQ, in which case Kruskal-Wallis with a *post hoc* Dunn’s multiple comparisons test was used. Results from GD17 were compared using an unpaired two-tailed t-test.

Overall, when one-way ANOVA was used and when there was a significant effect of group, a *post hoc* Tukey’s multiple comparisons test was performed. A p-value <0.05 was considered statistically significant.

## Supplementary information

Supplementary information.

## Data Availability

Data are available upon request.
