# Supplementary material for: Inner histopathologic changes and disproportionate zone volumes in foetal growth plates following gestational hypoglycaemia in rats
Source: Sci Rep. 2020 Mar 27;10:5609. doi: 10.1038/s41598-020-62554-2 (PMC7101337; doi:10.1038/s41598-020-62554-2)
Supplement: Supplementary file 1 — Supplementary information. [file 41598_2020_62554_MOESM1_ESM.pdf]

Supplementary information

Inner histopathologic changes and disproportionate zone volumes in foetal growth plates following gestational hypoglycaemia in rats

Jensen VFH[1,2,3], Mølck AM[1], Bøgh IB[1], Nowak J[1], Viuff BM[1], Rasmussen CLM[2], Pedersen L[2], Fels JJ[4], Madsen SH[4], McGuigan FE[3], Tveden-Nyborg P[2], Lykkesfeldt J[2], and Akesson KE[3]

Affiliation of authors

[1]Novo Nordisk A/S, Department of Toxicology, Safety Pharmacology and Pathology, Maaloev, Denmark; [2]University of Copenhagen, Department of Veterinary and Animal Sciences, Section for Experimental Animal Models, Copenhagen, Denmark; [3]Lund University, Department of Clinical Sciences Malmö and Skåne University Hospital, Department of Orthopedics, Malmö, Sweden; [4]Novo Nordisk A/S, Department of Research Bioanalysis, Maaloev, Denmark

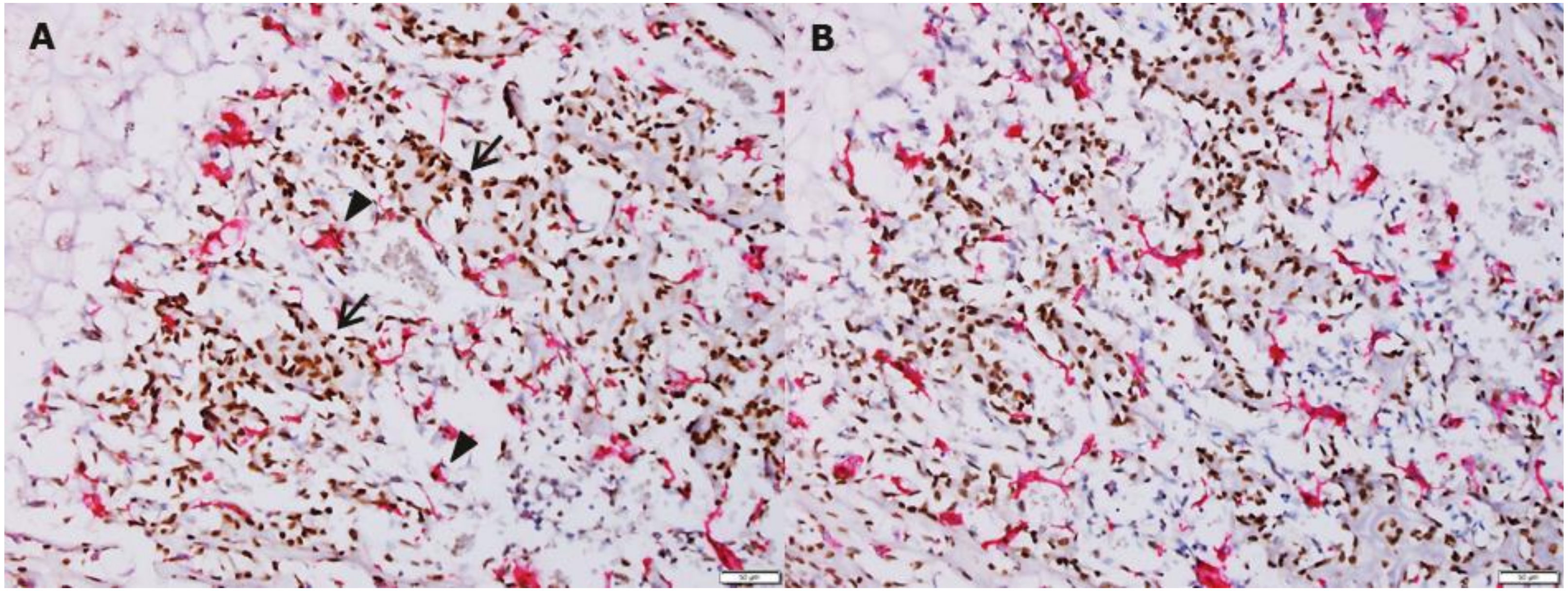

Figure S1 Representative pictures of immunohistochemical staining of osteoblasts and osteoclasts in foetal tibial primary ossification centres

Osteoclast cytoplasm (CD68, pink staining, arrowheads); osteoblast nucleus (Osterix, dark brown/black staining, arrows). (A) CTRL, (B) HI-*EoGest*. Ratio of osteoblast (nucleus): osteoclast (cytoplasm) volumes *was not affected by HI-infusion*. The number of sections assessed differed (due to varying foetal size; exclusion of some sections due to tissue folding; one HI-*EoGest* foetus was mistakenly omitted). Results are considered unlikely to be affected since ratios rather than absolute volumes were analysed. (Magnification x200)

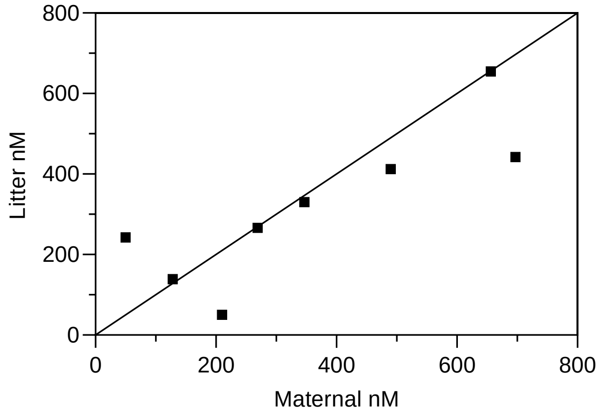

Figure S2. Correlation between plasma corticosterone level in individual HI-*EoGest* dams and their litters

Individual plasma corticosterone levels are from 8 HI-*EoGest* dams, where plasma level was also measured in the corresponding litters. Samples measured as <LLOQ were set to LLOQ (50 nmol/L), (1 dam and 1 litter). Maternal and litter levels were significantly correlated (Pearson r=0.834, p=0.0100).

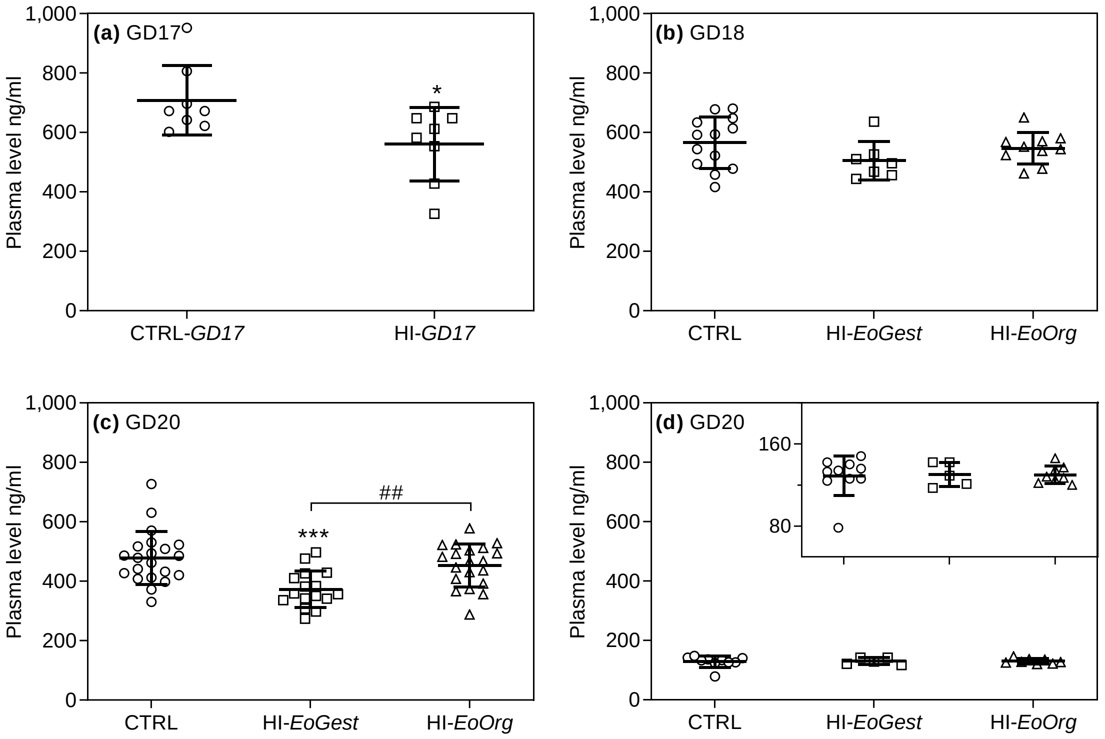

Figure S3. Plasma IGF-1 levels: maternal (a-c) and foetal (d)

Means±SD and individual values (symbols). (a) Maternal levels at sacrifice on GD17. (b) Maternal levels on GD18 (GD17+24h). (c) Maternal levels at sacrifice on GD20. (d) Foetal litter levels on GD20.

INSERT: Enlarged scale. *p<0.05 and ***p<0.001 versus CTRL-*GD17*/CTRL. ##p<0.01 HI-*EoOrg* versus HI-*EoGest*. (a): Unpaired two-tailed t-test, (b-d): One-way ANOVA with *post hoc* Tukey's multiple comparisons test.

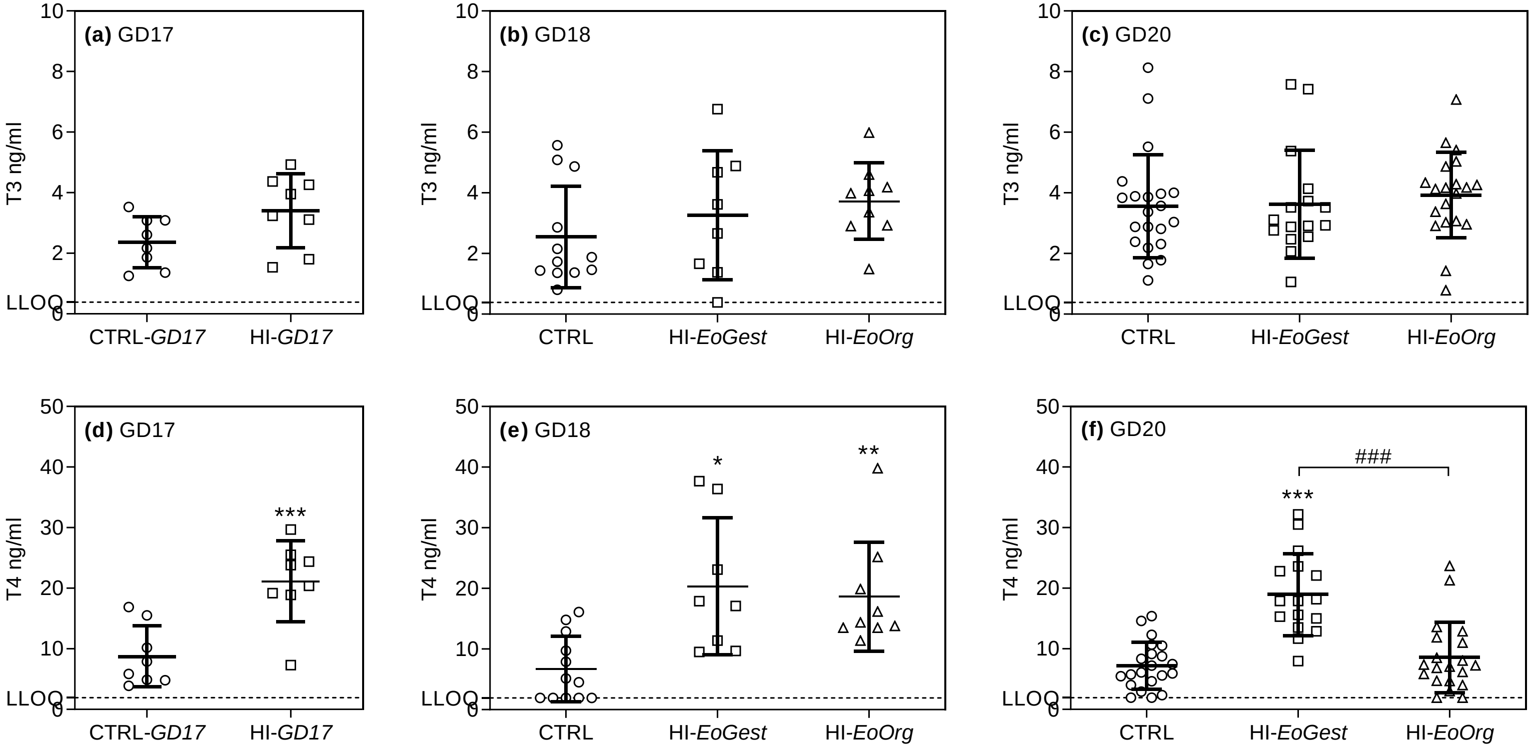

Figure S4 Maternal plasma levels of thyroid hormones: T3 (a-c) and T4 (d-f)

Means±SD, individual values. (a) T_3 level at sacrifice on GD17 (CTRL-GD17; n=8; HI-GD17, n=8). (b) T_3 level on GD18. (c) T_3 level on GD20. (d) T_4 level at sacrifice on GD17. (e) T_4 level on GD18. (f) T_4 level on GD20. Dotted line indicates LLOQ (0.391 ng/ml for $T_{3;}$ 1.95 ng/ml for T_4). Values measured as <LLOQ were set to LLOQ (T3: 1 HI-*EoGest* on GD18, T4: 5 CTRL on GD18 and 2 CTRL on GD20. All individual values are means of duplicate measurements, except 1 HI-*EoGest* sample on GD20 with limited volume. *p<0.05, **p<0.01, ***p<0.001 versus CTRL. $^{###}$p<0.001 HI-*EoOrg* versus HI-*EoGest*. (a+d): Unpaired two-tailed t-test, (b+c) and (e+f): One-way ANOVA with *post hoc* Tukey's test.

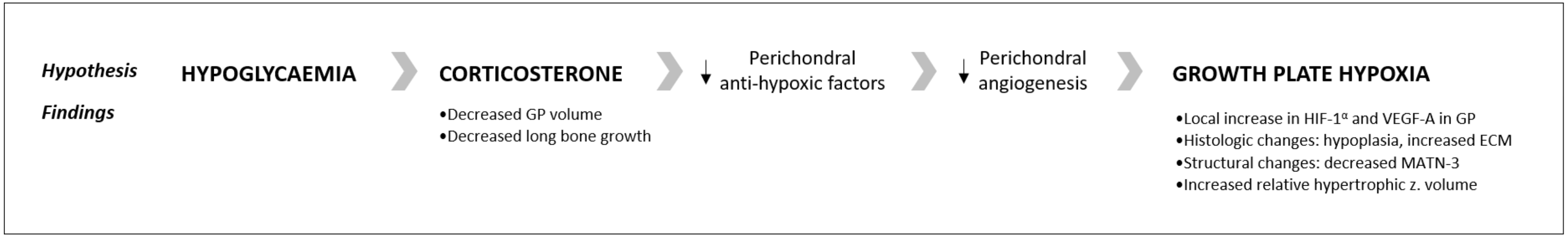

Figure S5 Hypothesised pathogenesis leading to foetal growth plate histopathology

Maternal and foetal plasma corticosterone levels increased during insulin-induced hypoglycaemia. We propose that corticosterone-induced hypoxia in growth plates is the main effector for the observed results.

Abbreviations: GP, growth plate; z, zone.

Immunohistochemistry Protocols

STAINING FOR COLLAGENS IN GROWTH PLATES

Pre-Treatment: Collagen II: dewaxed sections were subjected to 5 min. of digestion with proteinase K (S3020, Dako Denmark A/S, Glostrup, Denmark), 10 min. of resting, and rinsed in running water for 5 min.

Collagen X***:*** deparaffinised sections were heated in target retrieval solution (pH6, S2031, Dako Denmark A/S) for 15 min. in a microwave, followed by 10 min. resting and rinsing in running water for 5 min.

Staining Protocol: Endogenous peroxidase activity was blocked by incubation with Dako REAL Peroxidase-Blocking Solution (S2023, Dako Denmark A/S, Glostrup, Denmark) for 10 min., followed by rinsing with tris-buffered saline/Tween 20 0.01% (TBST) (AMPQ40829.5000, Ampliqon A/S, Odense, Denmark/P2287, Sigma-Aldrich Denmark A/S, Brøndby, Denmark) for 3 min. Sections were pre-incubated with TBST/5% BSA (A8677, Sigma-Aldrich Denmark A/S) for 30 min., which was then shaken off, followed by incubation for 60 min with a primary rabbit polyclonal antibody (*Collagen II*: 1:200, ab34712, abcam, Cambridge, UK. *Collagen X:* 1:600, orb10444, Biorbyt Ltd., Cambridgeshire, UK). Following a 2x 5 min. rinse with TBST, sections were incubated for 30 min. with the anti-rabbit labelled polymer BrightVision Poly-HRP-Anti Rb (DPVR HRP, Immunologic B. V., Duiven, Netherlands). After 3x 3 min. of rinsing with TBST, followed by 5 min. incubation with Liquid DAB+ Substrate Chromogen System (K3468, Dako Denmark A/S), sections were rinsed in running water for 5 min., a Mayer's haematoxylin (MHS80, Sigma-Aldrich Denmark A/S) counter-stain applied (45 sec.), and then rinsed with running water, dried on a hot plate and mounted in PERTEX (5500552, International Medical Products, Brussels, Belgium).

All incubations were at room temperature. Specificity of the collagen X antibody was confirmed by blocking of the signal by pre-incubation of the primary antibody with the corresponding immunogen peptide (orb374717, Biorbyt Ltd.). As the Collagen II antibody was raised against human and bovine cartilage, specificity was confirmed using rat lung tissue as a positive control for cartilage specific staining.

STAINING FOR OSTEOBLASTS AND OSTEOCLASTS IN THE PRIMARY OSSIFICATION CENTER

Staining Protocol: Deparaffinised sections were incubated with antigen retrieval TEG (pH 9) buffer (AMPQ17020, Ampliqon A/S) overnight at 60ºC, washed in demineralised water, then rinsed with TBST for 5 min., incubated with Dual Enzyme block (S2023, Dako Denmark A/S) for 10 min., 5 min. of TBST rinsing, then

blocked with 3% TBS/BSA solution for 30 min., followed by incubation with a primary rabbit polyclonal antibody against osterix (1:3500, ab22552, abcam) for 30 min (*for osteoblasts*).

Following 2 x 5 min. of TBST rinsing, sections were incubated for 30 min. with BrightVision Poly-HRP-Anti Rb (as above). After 2 x 5 min. of TBST rinsing, sections were incubated with a primary mouse monoclonal antibody against CD68 (1:1000, ab31630, abcam) for 30 min (*for osteoclasts*). Hereafter, sections were rinsed with TBST for 2 x 5 min. and incubated with Brightvision GaM-AP+20% rat serum (DPVM110AP, Immunologic B. V. and R9759, Sigma-Aldrich Denmark A/S) for 30 min., then with Permanent Red (K0640, Dako Denmark A/S) for 15 min., followed by 5 min. of rinsing with TBST, and 5 min. with demineralised water before applying a haematoxylin counter-stain (45 sec.). Sections were then incubated in tap water for 5 min., demineralised water for 5 min. and dried on a hot plate at 45ºC for about 5 min., before leaving slides at room temperature overnight. Slides were mounted in PERTEX (as above).

All incubations were at room temperature unless otherwise indicated. Specificity of the *osterix* antibody was confirmed by blocking of the signal by pre-incubation of the primary antibody with the corresponding immunogen peptide (ab24390, abcam). The CD68 antibody was raised against rat spleen cells, therefore, antibody specificity was confirmed using rat spleen tissue as positive control. There was no overlap between osterix and CD68 positive cells in tibial tissue sections.
